# Intradural extramedullary tumor in the stenotic cervical spine resected through open-door laminoplasty with hydroxyapatite spacers: report of two cases

**DOI:** 10.1186/s12893-018-0372-9

**Published:** 2018-06-11

**Authors:** Naohisa Miyakoshi, Daisuke Kudo, Michio Hongo, Yuji Kasukawa, Yoshinori Ishikawa, Yoichi Shimada

**Affiliations:** Department of Orthopedic Surgery, Akita University Graduate School of Medicine, 1-1-1 Hondo, Akita, 010-8543 Japan

**Keywords:** Cervical spine, Hydroxyapatite spacer, Laminoplasty, Spinal cord tumor

## Abstract

**Background:**

Safe excision of spinal cord tumors depends on sufficient visualization of the tumor and surrounding structures. In patients with spinal cord tumor adjacent to a stenotic spinal canal, extensive bony decompression proximal and distal to the tumor should be considered for safer excision of the tumor. Extensive wide laminectomy is one choice for such cases, but postoperative problems such as kyphotic deformity remain a concern.

**Case presentation:**

A 76-year-old man and a 60-year-old woman presented with symptomatic intradural extramedullary spinal cord tumors in the cervical spine. Both patients showed a combination of spondylotic changes in the cervical spine and stenotic condition at the level of the tumor. Both tumors were successfully resected through open-door laminoplasty with hydroxyapatite (HA) spacers, with the tumor located on the side of the laminoplasty. Histological diagnosis was schwannoma for both tumors. HA spacers completely bonded to the host bone and did not interfere with postoperative magnetic resonance imaging (MRI) of the inside of the spinal canal. Cervical spine alignment was maintained at the final follow-up of 6 years in both cases.

**Conclusion:**

Laminoplasty with HA spacers enabled successful tumor extirpation, reliable MRI follow-up after surgery, and maintenance of normal cervical spine alignment. Laminoplasty with HA spacers represents a good option for the treatment of cervical spinal cord tumor in patients combined with spinal stenosis.

## Background

Safe excision of spinal cord tumors depends on sufficient visualization of the tumor and surrounding structures. If patients with spinal cord tumor also show a stenotic spinal canal at and/or adjacent to the segment affected by the tumor, more extensive bony decompression proximal and distal to the tumor should be considered to allow safer excision of the tumor. The cervical spine often shows stenosis of the spinal canal due to spondylotic changes, including osteophyte formation and protrusions of the intervertebral discs with advancing age. One study showed cervical intervertebral disc protrusion with spinal cord compression in 7.6% of asymptomatic volunteers, with higher prevalence among the elderly [1].

A posterior approach is commonly used for intradural extramedullary tumors other than anterior lesions. Although laminectomy is a standard surgical technique, post-laminectomy kyphosis from disruption of the posterior elements can be anticipated, especially in the cervical spine [2]. Various types of laminoplasty for decompression of myelopathy have been developed to maintain the dorsal integrity of the cervical spine [3–5]. To prevent post-laminectomy kyphosis, additional instrumented posterior fusion is one option, but studies have shown that laminoplasty reduces complications such as infection, C5 palsy and pseudarthrosis compared with laminectomy and fusion [6–9].

Open-door laminoplasty has been widely used for the treatment of cervical spondylotic myelopathy [3] and other pathologies causing myelopathy, including spinal cord tumor [10]. This procedure can provide access to the tumor comparable to conventional laminectomy, while preserving posterior structures of the cervical spine. Furthermore, using hydroxyapatite (HA) spacers in laminoplasty offers the advantage of this material in terms of the ability to bond to bone [11].

Here, we report our experience with two cases of cervical intradural extramedullary tumor successfully resected through open-door laminoplasty with HA spacers. Both patients showed an underlying spondylotic stenotic condition in the cervical spine, and were followed-up for more than 5 years postoperatively. Advantages of spinal cord tumor resection with laminoplasty using HA spacers in such patients are discussed.

## Case presentation

### Case 1

**Fig. 1:**
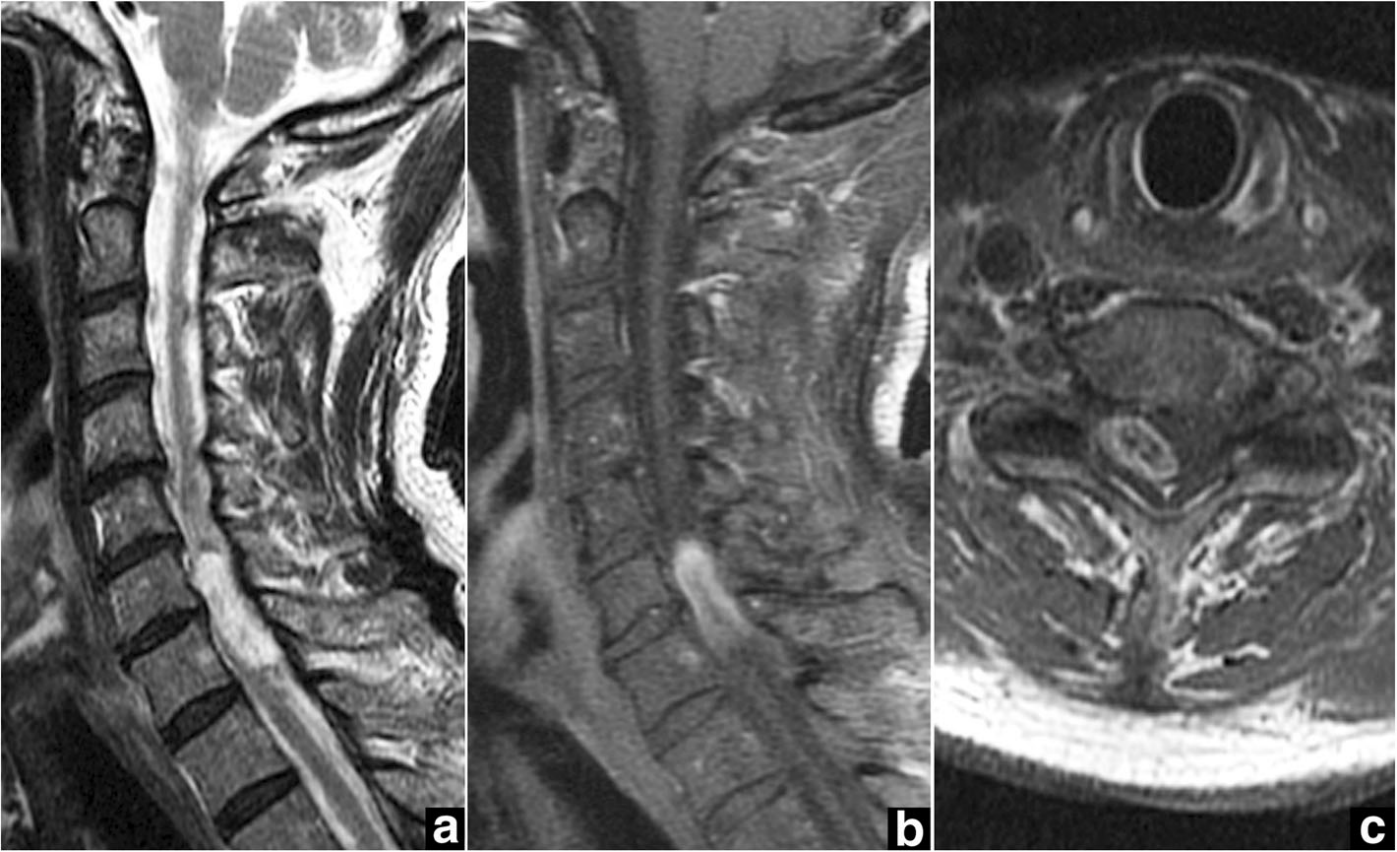
Case 1. Preoperative magnetic resonance imaging (MRI) of the cervical spine. **a** Sagittal T2-weighted MRI shows the intradural extramedullary spinal cord tumor with signal hyperintensity at C6-C7 and spinal canal stenosis at C4-C7. **b**, **c** Gadolinium-enhanced sagittal MRI (**b**) and axial MRI (**c**) at the C6-C7 level show a heterogeneously enhanced tumor located dorsally and to the right of the spinal cord

A 76-year-old man presented to our hospital with a 4-week history of progressive pain in the neck and right upper extremity, spastic gait, and numbness in both legs. Physical examination showed normal muscular strength, but hyperactive deep tendon reflexes in both legs. Cervical spine X-ray demonstrated degenerative spondylosis. Magnetic resonance imaging (MRI) revealed an intradural extramedullary spinal cord tumor at the C6-C7 level, appearing hypointense on T1-weighted imaging and hyperintense on T2-weighted imaging (Fig. 1a), with heterogeneous gadolinium enhancement (Fig. 1b, c). The tumor was located posterolateral to the spinal cord, on the right. Mild spinal canal stenosis was also evident at the C4-C7 level.

**Fig. 2:**
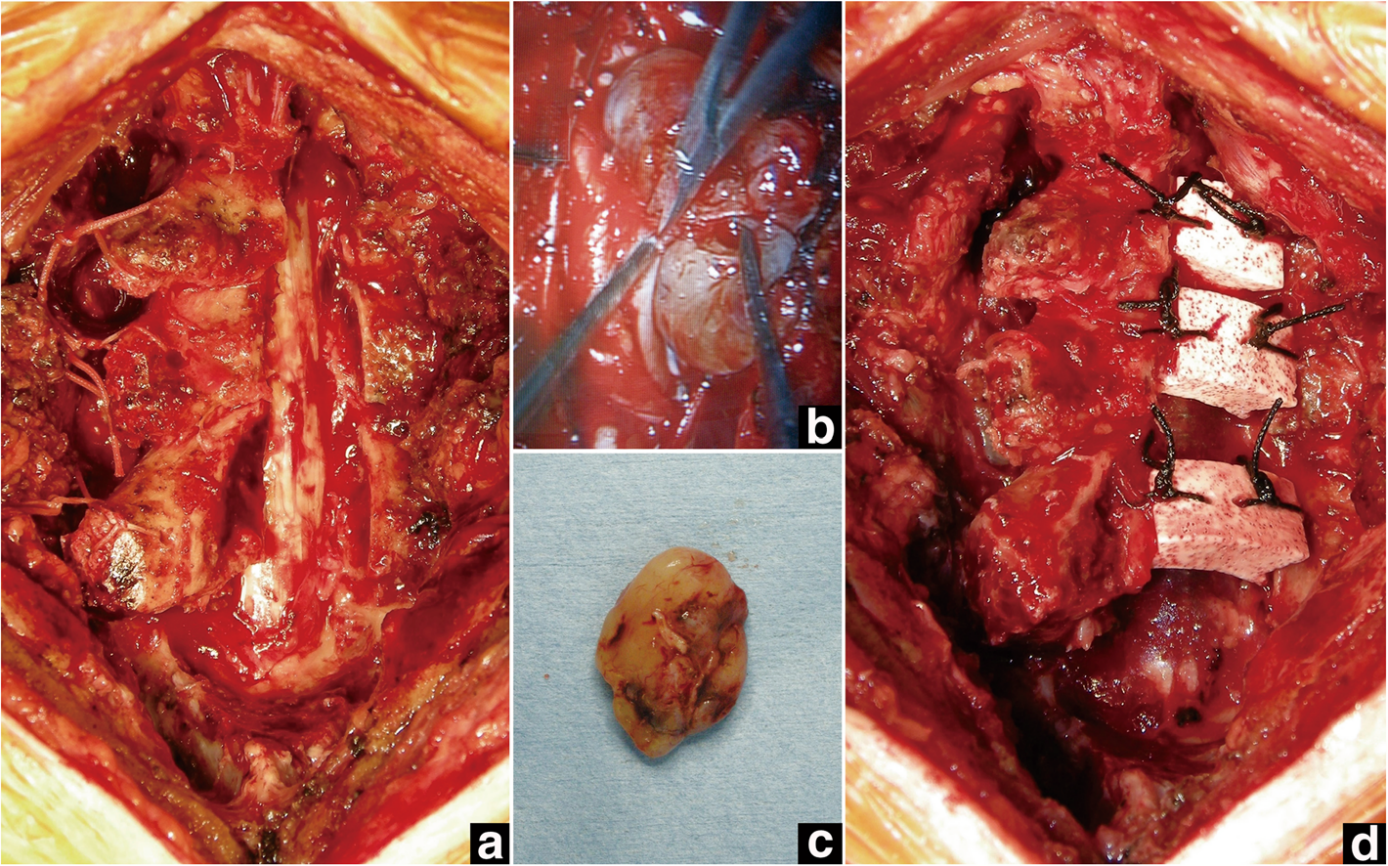
Case 1. Intraoperative photographs. **a** Laminae from C5 to C7 are fully opened with the right side as the opening side. **b** After opening the dura, the tumor is detached from the spinal cord microscopically. **c** The tumor is totally extirpated. **d** After extirpation of the tumor and dural closure, HA spacers are placed between the right-side laminae and lateral mass from C5 to C7

The intradural extramedullary tumor was resected through open-door laminoplasty at C5-C7. After midline skin incision, the tip of the C7 spinous process (approx. 1 cm in length) was cut in half with an oscillating bone saw. The nuchal ligament was sharply and longitudinally dissected by scalpel and retracted bilaterally along with the tip of the C7 spinous process. The open-door laminoplasty was performed by creating bilateral gutters at the junction of the laminae and the medial aspect of the lateral mass. In this case, the right side was the opening side, with the left side gutter acting as the hinge (Fig. 2a). The laminae from C5 to C7 were fully opened, and the position was kept with stitches between the laminae and paravertebral muscles. Following the opening of the laminae, the dura mater was opened under microscopy, and the tumor was totally extirpated under motor-evoked potential (MEP) monitoring (Fig. 2b, c). After tumor resection, HA spacers were placed between the right-side laminae and the lateral mass from C5 to C7 (Fig. 2d). The bilaterally opened nuchal ligament with the tips of the C7 spinous processes was then tightly closed. The histological diagnosis of the tumor was schwannoma. The postoperative course was uneventful and the patient recovered completely, without any symptoms.

**Fig. 3:**
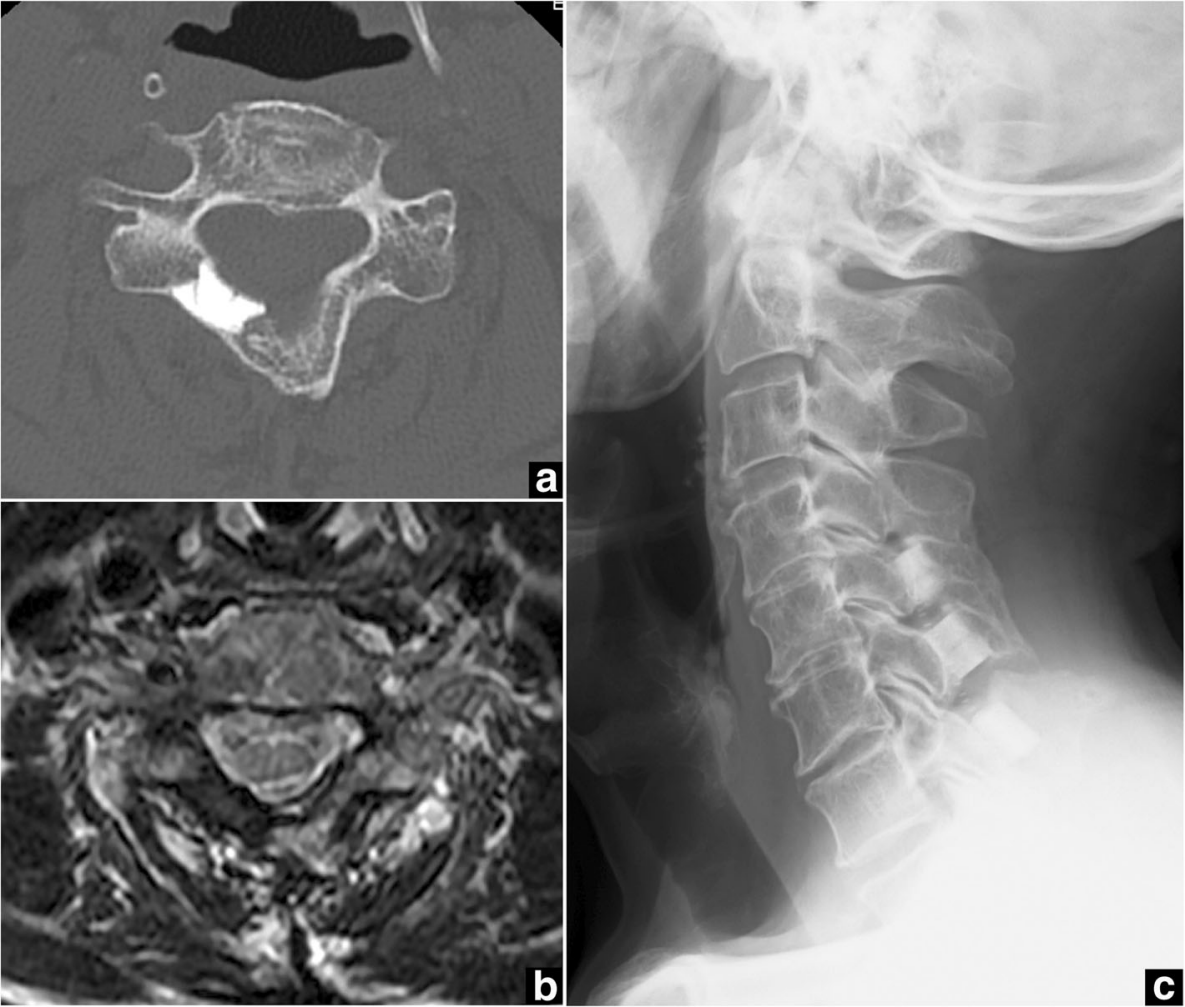
Case 1. Postoperative computed tomography (CT), magnetic resonance imaging (MRI), and plain X-ray of the cervical spine. **a** Axial CT at the C6 level obtained 5 years after surgery demonstrates a newly created spinal canal with HA spacers bonded to host bone. **b** Axial MRI at the C6 level obtained 5 years after surgery shows no tumor recurrence with good visualization inside the spinal canal. **c** Lateral plain X-ray obtained 6 years after surgery shows normal sagittal alignment

On the 5-year follow-up CT, all C5-C7 spacers had bonded directly to the host bone, creating a wide new canal space (Fig. 3a). MRI at 5 years postoperatively achieved good visualization of the spinal cord under the HA spacers, with no tumor recurrence (Fig. 3b). Cervical spine X-ray at 6 years postoperatively showed normal alignment and no kyphotic deformity (Fig. 3c).

### Case 2

A 60-year-old woman with a 2-year history of numbness in both hands and legs was referred to our hospital after experiencing a marked deterioration in walking ability. Neurological examination showed weakness in both legs (grades 3–4/5 on manual muscle testing). Both hands had lost dexterity, and deep tendon reflexes were hyperactive in both legs.

**Fig. 4:**
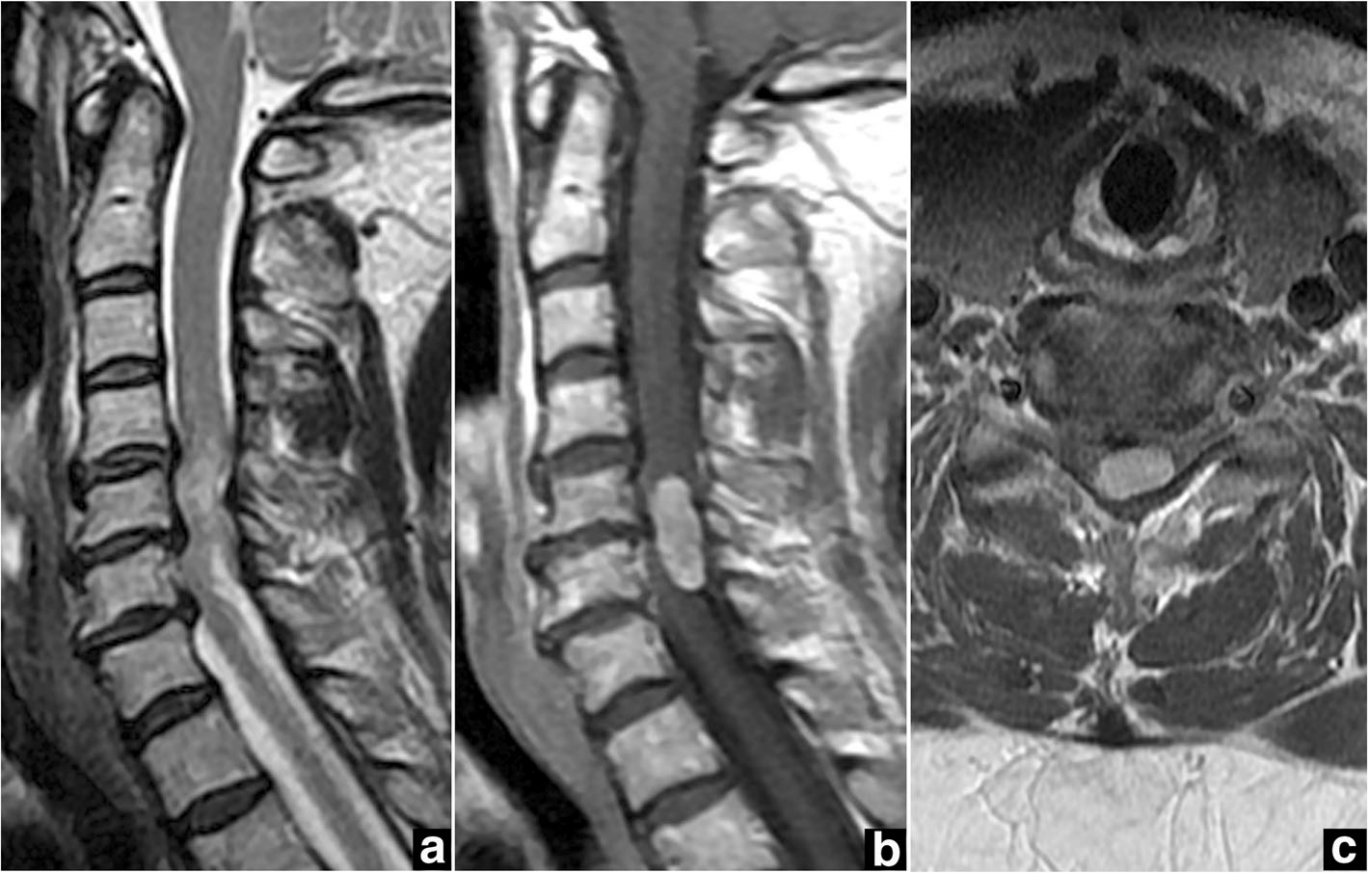
Case 2. Preoperative magnetic resonance imaging (MRI) of the cervical spine. **a** Sagittal T2-weighted MRI shows the intradural extramedullary spinal cord tumor with heterogeneous signal hyperintensity at C5-C6 and spinal canal stenosis at C4-C7. **b**, **c** Gadolinium-enhanced sagittal MRI (**b**) and axial MRI (**c**) at the C5-C6 level show homogeneous enhancement of a tumor located dorsally and to the left of the spinal cord

Cervical spine X-ray demonstrated degenerative spondylosis. Intradural extramedullary spinal cord tumor was identified on MRI at the C5-C6 level, appearing hypointense on T1-weighted imaging and heterogeneously hyperintense on T2-weighted imaging (Fig. 4a), with homogeneous gadolinium enhancement (Fig. 4b, c). The tumor was located posterolateral to the spinal cord, on the left. Spinal canal stenosis was seen at the C4-C7 level.

The intradural extramedullary tumor was resected through open-door laminoplasty of C5-C7 using HA spacers. The surgical procedure was the same as described for Case 1. In Case 2, the tumor was located to the left and posterior to the spinal cord, so the left-side laminae from C5 to C7 were opened with the right-side gutter as a hinge. The tumor was totally resected without complications under MEP monitoring. The histological diagnosis of the tumor was schwannoma. The postoperative course was uneventful and the patient recovered all neurological functions within 3 weeks.

**Fig. 5:**
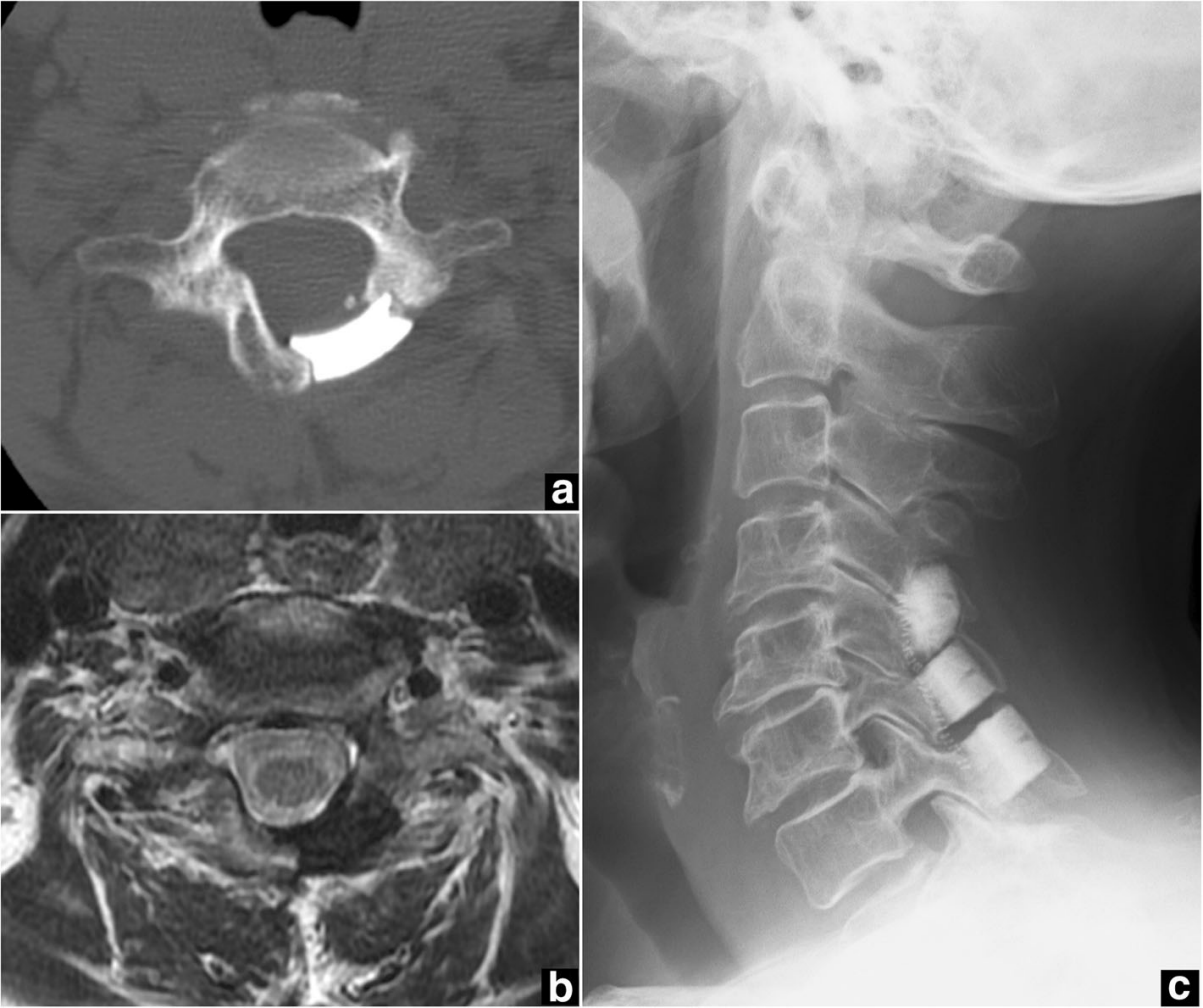
Case 2. Postoperative computed tomography (CT), magnetic resonance imaging (MRI), and plain X-ray of the cervical spine. **a** Axial CT at the C5 level 1 year after surgery demonstrates a newly created spinal canal with HA spacers bonded to host bone. **b** Axial MRI at the C5 level obtained 5 years after surgery shows no tumor recurrence with good visualization inside the spinal canal. **c** Lateral plain X-ray obtained 6 years after surgery shows normal sagittal alignment

On follow-up CT performed 1 year after surgery, bone on the opened side appeared directly bonded to the HA spacers (Fig. 5a). MRI at 5 years postoperatively achieved good visualization of the spinal cord under the HA spacers, with no tumor recurrence (Fig. 5b). Cervical spine X-ray at 6 years after surgery showed normal alignment (Fig. 5c).

## Discussion and conclusions

Sufficient intraoperative visualization is essential for the surgical treatment of spinal cord tumors. Several approaches for the resection of spinal cord tumors have been reported, including bilateral total laminectomy, hemi-laminectomy, laminoplasty with spacers or plates, and recapping laminoplasty [10, 12–15]. Bilateral total laminectomy has normally been used as a standard procedure. This technique provides a wider visual field enabling safer management of the tumor, and thus preventing neurological complications. However, this procedure is reportedly associated with several issues, including postoperative instability and spinal kyphosis [12, 16]. A biomechanical study demonstrated that loss of the posterior ligamentous and bony elements caused a forward shift in the weight-bearing axis and a subsequent increase in force on the anterior vertebral body [12]. Moreover, postoperative denervation and atrophy of the posterior cervical muscles and injury to the facet joints have been demonstrated to worsen such deformity [12].

To prevent postoperative spinal deformity, laminoplasty was developed as an alternative to laminectomy. In fact, for the treatment of cervical degenerative myelopathy, postoperative kyphosis has been reported to occur in only 5–7% of patients after laminoplasty, compared to 14–47% of patients after laminectomy alone [16]. Furthermore, in terms of spinal cord tumor resection, Montano et al. [10] reported the effects of laminoplasty for intradural spinal cord tumor, compared with laminectomy in their case series, and concluded that laminoplasty was not associated with any new onset of spinal deformities, but was associated with a lower rate of spinal deformity progression after intradural intra- or extramedullary tumor resection. In the current report, although the precise contribution provided by laminoplasty to the prevention kyphosis is unclear, neither of our cases showed progression of kyphosis for more than 5 years of follow-up.

The surgical technique for cervical laminoplasty can be broadly divided into two methods. The first technique is open-door laminoplasty [17], as used in the current cases. The second technique is double-door laminoplasty, which is performed by splitting the spinous processes sagittally [18]. In the present cases, since the tumors were located asymmetrically and associated with spinal canal stenosis, the open-door method was needed to ensure safe removal of the tumors and concurrent expansion of the narrow spinal canal. If we had tried to excise these unilaterally located tumors with double-door laminoplasty, safe resection would have been difficult.

Since the late 1990s, metal mini-plates have been used for cervical laminoplasty as an alternative to sutures, anchors, and local spinous process autografts to provide more rigid, lasting fixation [5]. However, HA spacers have commonly been used for laminoplasty in Japan. Laminoplasty using HA spacers seems to offer several advantages compared with that using metal implants in our clinical setting. First, since follow-up with CT or MRI is indispensable in tumor cases to identify tumor recurrence after surgery, laminoplasty using HA spacers can provide sufficient intra-spinal canal information, as shown in our cases, because HA does not result in any of the artifacts usually seen with metal implants. Moreover, metal implants interfere with the beams when radiotherapy is needed after surgery [19]. Posterior metal implants resulted in a 5–7% decrease in the radiation dose delivered to the spinal canal in sawbone models [20]. Second, HA spacers have been histologically confirmed to show the ability to bond directly to bone, along with bone ingrowth into the spacer [11]. Third, the surgical procedure for laminoplasty using HA seems less technically demanding. Operative time, operative blood loss and perioperative complication rate did not differ significantly between residents and teaching neurosurgeons [21]. However, non-union between the spacers and host bone may occur if inadequate contact is achieved [17]. One study showed a mean non-union rate for HA spacers of 17–21%, and an average breakage rate of 21–24% at a minimum of 10 years of follow-up after double-door laminoplasty for compressive cervical myelopathy [22]. However, neither non-union nor breakage of HA spacers was related to restenosis of an enlarged cervical canal [22]. Those data for open-door laminoplasty with HA spacers remain unclear. In the present cases, HA spacers completely bonded to host bone after open-door laminoplasty, and normal cervical spinal alignment was maintained for more than 5 years of follow-up.

In conclusion, we reported two cases of cervical intradural extramedullary tumor in patients with spinal stenosis successfully treated through open-door laminoplasty using HA spacers. Laminoplasty with HA spacers enabled successful tumor extirpation, very reliable follow-up with MRI after surgery, and maintenance of normal cervical spine alignment for more than 5 years. Open-door laminoplasty with HA spacers represents a good option for the treatment of spinal cord tumors in patients with spinal stenosis.

## Abbreviations

CT: Computed tomography
HA: Hydroxyapatite
MRI: Magnetic resonance imaging
